# Recent advances and challenges in plant viral diagnostics

**DOI:** 10.3389/fpls.2024.1451790

**Published:** 2024-08-13

**Authors:** Aizada Kanapiya, Ulbike Amanbayeva, Zhanar Tulegenova, Altyngul Abash, Sayan Zhangazin, Kazbek Dyussembayev, Gulzhamal Mukiyanova

**Affiliations:** ^1^ Department of Biotechnology and Microbiology, L.N. Gumilyov Eurasian National University, Astana, Kazakhstan; ^2^ Laboratory of Biodiversity and Genetic Resources, National Center for Biotechnology, Astana, Kazakhstan; ^3^ Scientific Center "Agrotechnopark", Shakarim University, Semey, Kazakhstan

**Keywords:** plant virus, disease diagnostics, biosensor, sequencing technologies, CRISPR-Cas

## Abstract

Accurate and timely diagnosis of plant viral infections plays a key role in effective disease control and maintaining agricultural productivity. Recent advances in the diagnosis of plant viruses have significantly expanded our ability to detect and monitor viral pathogens in agricultural crops. This review discusses the latest advances in diagnostic technologies, including both traditional methods and the latest innovations. Conventional methods such as enzyme-linked immunosorbent assay and DNA amplification-based assays remain widely used due to their reliability and accuracy. However, diagnostics such as next-generation sequencing and CRISPR-based detection offer faster, more sensitive and specific virus detection. The review highlights the main advantages and limitations of detection systems used in plant viral diagnostics including conventional methods, biosensor technologies and advanced sequence-based techniques. In addition, it also discusses the effectiveness of commercially available diagnostic tools and challenges facing modern diagnostic techniques as well as future directions for improving informed disease management strategies. Understanding the main features of available diagnostic methodologies would enable stakeholders to choose optimal management strategies against viral threats and ensure global food security.

## 1 Introduction

Plant pathogens are diverse groups of microorganisms that cause various diseases in plants, which can result in serious economic losses in agriculture. After fungi, viruses are the second most prevalent plant pathogens (Wang et al., 2023). These microscopic parasites are composed of small particles containing nucleic acid (either RNA or DNA) encased in a protein shell. Typically, viruses measure just a few nanometers in size and infect plants, causing a range of diseases and crop damage (Kovalskaya and Hammond, 2014; Manjunatha et al., 2022; Huang et al., 2023).

Viruses can infect all kinds of plants leading to huge economic damage worth many billions of dollars annually. They are the main pathogens causing both new and recurrent plant diseases worldwide, and cause damage to both natural vegetation and cultivated plants (Ghorbani et al., 2023; Huang et al., 2023). For instance, it was revealed the spread and emergence of *Potato virus Y* strains, including strains that cause economically important diseases of tobacco, tomatoes, and peppers, as well as the fact that the virus continues to develop with the relatively recent emergence of new damaging recombinant strains (Torrance and Talianksy, 2020). This evolution of *Potato virus Y* strains presents significant challenges for disease management and highlights the importance of continuous monitoring and adaptation of control strategies. Plant viruses affect plant life processes such as photosynthesis, metabolism and growth, which can ultimately lead to characteristic symptoms of the disease, such as yellowness of the leaves, deformations of plants, the formation of spots and blisters on the leaves, as well as tissue death (Jiang and Zhou, 2023).

As part of plant science, the study of plant viral diagnostics helps to develop disease control strategies, such as selecting resistant plant varieties, applying chemical and biological preparations to prevent the spread of viruses. Chemical control of plant viruses has been a significant aspect of integrated disease management programs. Particularly, insecticides are mostly employed for their convenience and effectiveness in preventing virus transmission by vectors, and this is also because direct control measures are usually unsuccessful against viruses (Garzo et al., 2020). However, it poses environmental and health risks despite its occasional effectiveness (Pandit et al., 2022). These risks underline the need to find alternative methods to combat viral diseases of plants. Accurate identification of pathogens is a key component of controlling plant diseases, as early detection allows for effective measures to control and prevent their spread (Kalimuthu et al., 2022). Conventional diagnostic systems include various methods such as selective cultural, immunological (Edwards and Cooper, 1985) and molecular (Olmos et al., 2007). In addition to detecting viruses, these methods determine their type and quantity, which is useful for developing management strategies and controlling plant diseases. Furthermore, there are high-performance next-generation sequencing platforms that provide powerful tools for the identification and surveillance of viral infections.

## 2 Conventional methods for detection of plant viruses

In order to develop and apply protective agents against viral infections of plants, it is necessary to have detection methods with high sensitivity and specificity that will be available for practical use in agricultural conditions (Devi et al., 2024). Conventional methods for detecting plant viruses can achieve a balance between the reliability of the results and the practicality of their application. The main tools for routine screening and diagnosis of viral infections are enzyme-linked immunosorbent assay (ELISA) and amplification-based molecular approaches.

### 2.1 Enzyme-linked immunosorbent assay

ELISA is a convenient and sensitive tool for detecting the presence of viral agents and their quantification in plant tissues (Bramhachari et al., 2019). The method is based on the specific interaction of antibodies with proteins, in case of viral detection, mainly with the capsid protein of the target virus. If the target virus is present in the sap of the plant, it will interact with primary antibodies. After removing the unrelated primary antibodies, a reporter molecule, usually an enzyme, is added to the secondary antibodies, which allows the virus to be detected by forming a chromogenic product on the substrate (Nascimento et al., 2017).

Secondary antibodies target the permanent region of the primary antibody. For example, for primary antibodies derived from rabbits and directed against a viral antigen, enzyme-linked anti-rabbit IgG obtained from other mammals such as cattle, horse and goat can be used. In case with plant viral detection, plant juice extract is added to the wells of the microtiter tablet, then antibodies are added after incubation, followed by rinsing. If the virus of interest is present in the plant, it will bind to antibodies. Any unbound extract is washed off before adding a secondary antibody that recognizes the primary antibody (Abd El-Aziz, 2019).

**Figure 1:**
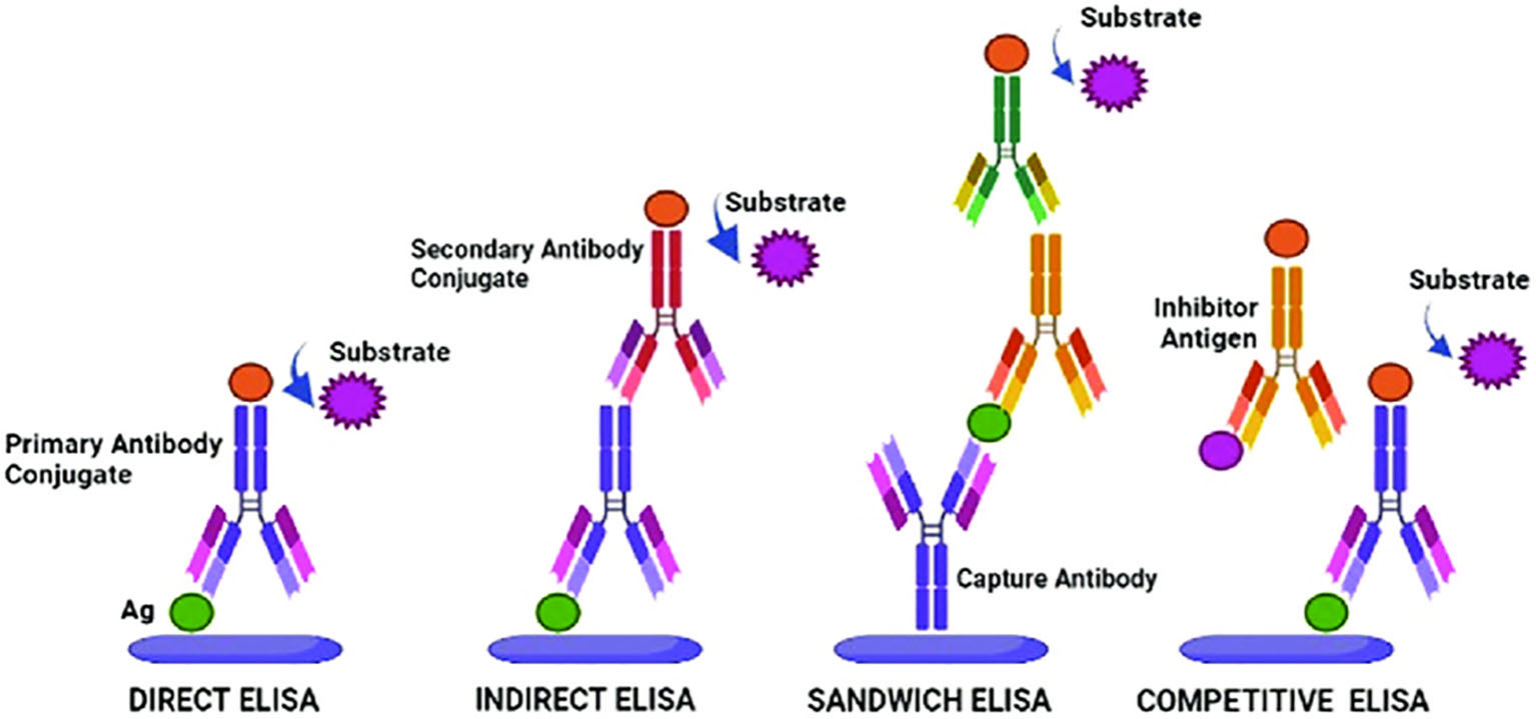
ELISA types and their working principles. Adopted with permission from Khan et al. (2023).

According to the method of binding antibodies and antigens, as well as the level of sensitivity and specificity, several main types of ELISA have been developed (**Figure 1**). Direct ELISA uses one type of antibodies associated with the enzyme, therefore it is convenient and fast, but may be less sensitive. Indirect ELISA offers increased sensitivity and specificity using two antibodies, as well as sandwich ELISA, which is the best choice for complex antigens with multiple epitopes. Competitive ELISA is effective for the accurate quantification of antigens, especially in the case of low concentrations or small sizes, since it is based on competition between labeled and unlabeled antigens for binding to an antibody (Khan et al., 2023).

ELISA-based methods, such as direct tissue blot immunoassay, double antibody sandwich ELISA, and tissue-print ELISA are the most popular in viral identification (Abd El-Aziz, 2019). There are also several modifications of ELISA that offer improved methodologies and diverse applications in various fields. The plate-trapped antigen ELISA demonstrated high sensitivity and specificity for detecting viruses that cause significant economic losses in important Brazilian crops. The kit was able to detect six viral species from the genera *Comovirus*, *Cucumovirus*, *Potyvirus*, and *Sobemovirus* in infected plant tissues (Nascimento et al., 2017). A few examples of ELISA techniques developed for plant viral diagnostics are summarized in **Table 1**.

**Table 1:** ELISA tests developed for plant viral disease diagnostics.

Technique	Crop	Pathogen	Ref.
Dot-ELISA	Sugarcane Maize Potato Tomato	*Sorghum mosaic virus* *Maize chlorotic mottle virus* *Maize dwarf mosaic virus* *Potato virus S* *Potato virus M* *Tomato mottle mosaic virus*	Faccioli and Colombarini (1996) Chen et al. (2020) Li et al. (2021) Javaran et al. (2021)
Plate-trapped antigen ELISA	Squash Cucumber Cowpea Zucchini Papaya	*Squash mosaic virus* *Cowpea severe mosaic virus* *Cucumber mosaic virus* *Cowpea aphidborne mosaic virus* *Zucchini yellow mosaic virus* *Papaya lethal yellowing virus* *Zucchini lethal chlorosis virus*	Nascimento et al. (2017) Ramos et al. (2018)
Triple antibody sandwich ELISA	Cucumber Pepper Tobacco Tomato Potato Canary Carrizo citrange Cassava	*Cucumber mosaic virus* *Pepper mild mottle virus* *Tobacco mosaic virus* *Odontoglossum ringspot virus* *Tomato mosaic virus* *Ribgrass mosaic virus* *Potato mop-top virus* *Barley yellow dwarf virus* *Cereal yellow dwarf virus* *Citrus psorosis virus* *Sri Lankan cassava mosaic virus*	Arif et al. (1994) Martín et al. (2004) Yu et al. (2005) Ilbagi et al. (2008) Phatsaman et al. (2020) Charoenvilaisiri et al. (2021)
Double antibody sandwich ELISA	Potato Peanut Chrysanthemum Sorghum	*Potato virus Y* *Potato virus S* *Tomato spotted wilt virus* *Tomato black ring virus`* *Sugarcane streak mosaic virus*	Hema et al. (1999) Lai et al. (2021) Chinnaiah et al. (2022) Tessema et al. (2024)

ELISA is recognized as the world standard for detecting viruses in crops due to a number of advantages such as ease of implementation and sensitivity, which allows detecting low concentrations of viral samples. Moreover, it also offers versatility, which allows adapting to detect a wide range of plant viruses through the use of various antibodies (Mehetre et al., 2021). However, despite its many advantages, ELISA also has a few disadvantages that may limit its use in certain situations. The specificity of ELISA depends on the quality of the antibodies used in the analysis. Insufficient purity or specificity of antibodies can lead to false positive or false negative results, which reduces the reliability of diagnosis. The production of high-quality antibodies also requires significant costs and time (Tabatabaei et al., 2021). Despite this, ELISA remains the main method for routine screening and diagnosis of viral diseases in agriculture due to its balance between sensitivity, specificity, cost and ease of use.

### 2.2 Polymerase chain reaction

Molecular methods are one of the most promising approaches to the diagnosis of plant viruses. These methods are based on the analysis of nucleic acids of viruses, such as DNA and RNA, using various techniques of molecular biology (Mehetre et al., 2021). One of the key molecular methods is PCR, which allows to increase the number of certain DNA or RNA fragments. The PCR have been used for detection of many plant viruses such as *Banana bunchy top virus* (Wang et al., 2022), *Tobacco ringspot virus* (Lee et al., 2015), *Bean common mosaic virus* (Xu and Hampton, 1996), *Apple mosaic virus* (Nabi et al., 2023), *Papaya ringspot virus* (Hamim et al., 2018) and more (**Table 2**). Based on PCR, many modifications were developed, including reverse transcription PCR (RT-PCR), quantitative PCR (qPCR), nested PCR and multiplex PCR (Wang et al., 2022).

**Table 2:** Application of PCR-based molecular approaches in plant virus diagnostics.

Technique	Pathogen	Crop	References
qPCR	*Tobacco mosaic virus* *Cucumber mosaic virus* *Sugarcane bacilliform virus* *Sugarcane bacilliform IM virus* *Sugarcane bacilliform MO virus*	Tobacco Sugarcane	Feng et al. (2006) Sun et al. (2018) Ellis et al. (2020)
RT-qPCR	*Citrus tristeza virus* *Citrus leaf blotch virus* *Tobacco Mosaic Virus* *Barley yellow dwarf virus* *Cereal yellow dwarf virus*	Citrus plants Tobacco Barley	Balaji et al. (2003) Ruiz-Ruiz et al. (2007) Ruiz-Ruiz et al. (2009) Kokane et al. (2021b)
Nested PCR	*Prune dwarf virus*, *Prunus necrotic ringspot virus* *Apple mosaic virus* *Lettuce mosaic virus* *Squash vein yellowing virus*	Prune Apple Lettuce Squash	Moreno et al. (2007) Maliogka et al. (2010)
Multiplex PCR	*Banana streak Mysore virus* *Banana bunchy top virus* *Capsicum chlorosis orthotospovirus* *Chilli veinal mottle virus* *Large cardamom chirke virus Cucumber mosaic virus* *Pepper mild mottle virus* *Chilli leaf curl virus* *Artichoke Italian latent virus*	Banana Chili Cucumber Pepper Artichoke	Selvarajan et al. (2011) Minutillo et al. (2012) Devi et al. (2022)

PCR can detect infectious agents at the earliest stages of infection, which significantly increases the effectiveness of measures to combat plant diseases. It is also versatile, as it can be used to diagnose a wide range of viruses, bacteria and fungi, unlike ELISA, which requires different sets of antibodies for each individual pathogen. The high sensitivity of PCR makes it possible to detect low concentrations of DNA, which has a risk of leading to false positives (Venbrux et al., 2023).

Particularly, qPCR is more sensitive in detecting small concentrations of target viruses while significantly reducing detection time compared to other PCR methods. Its swifter pace is attributed to the elimination of the gel electrophoresis step required for confirmation, thereby lowering the risk of contamination (Hema and Konakalla, 2021). The qPCR, also known as real-time PCR, allows for the amplification and simultaneous quantification of specific DNA or RNA sequences. This method leverages the sensitivity and specificity of PCR, coupled with real-time measurement of the amplified product using fluorescent dyes or probes. Furthermore, the ability to quantify viral load precisely in infected plants makes qPCR particularly valuable for understanding the dynamics of virus infection and for making informed decisions in plant disease management.

qPCR is increasing its applications in plant virus diagnostics with many reported assays to detect viruses. Based on SYBR Green I, the qPCR and a nested RT-PCR were developed to detect *Potato mop-top virus*. The detection sensitivity was several times higher than in dot-blot hybridization and standard PCR analysis (Zhou et al., 2019). In another study, a SYBR Green-based RT-qPCR assay was developed for the detection of *Indian citrus ringspot virus* (Kokane et al., 2021a). The assay was highly specific to *Indian citrus ringspot virus*, showing no cross-reactivity with other citrus pathogens, and was about 100 times more sensitive than conventional RT-PCR. SYBR Green is based on the use of a fluorescent dye that binds to double-stranded DNA, whereas another TaqMan qPCR technique uses fluorescent probes to specifically identify target sequences. Real-time analysis of TaqMan qPCR was performed using a newly designed primer pair, and the assay was reproducible and could specifically detect *Banana streak virus* without cross-reaction with *Cucumber mosaic virus* and *Banana terminal virus* at the same time showing higher sensitivity than Endpoint PCR (Jie et al., 2017). Overall, qPCR offers several advantages over traditional PCR, including increased sensitivity, specificity, and the ability to quantify DNA in real-time (Venbrux et al., 2023).

### 2.3 Isothermal amplification methods

There is an increasing trend in the rapid development of molecular technologies, which have demonstrated effective advancements for diagnostics. PCR is the golden standard in molecular diagnostics due to its high sensitivity and specificity. However, PCR methods require three different temperature regimes, necessitating expensive equipment. Additionally, the complexity and stringent laboratory requirements limit the use of PCR in field conditions. As a result, several alternative isothermal amplification methods have been emerged. Many methods of isothermal amplification have been developed, the most well-known loop-mediated isothermal amplification (LAMP) and recombinase polymerase amplification (RPA) have widely been used for plant pathogen detection (Moon et al., 2022).

**Figure 2:**
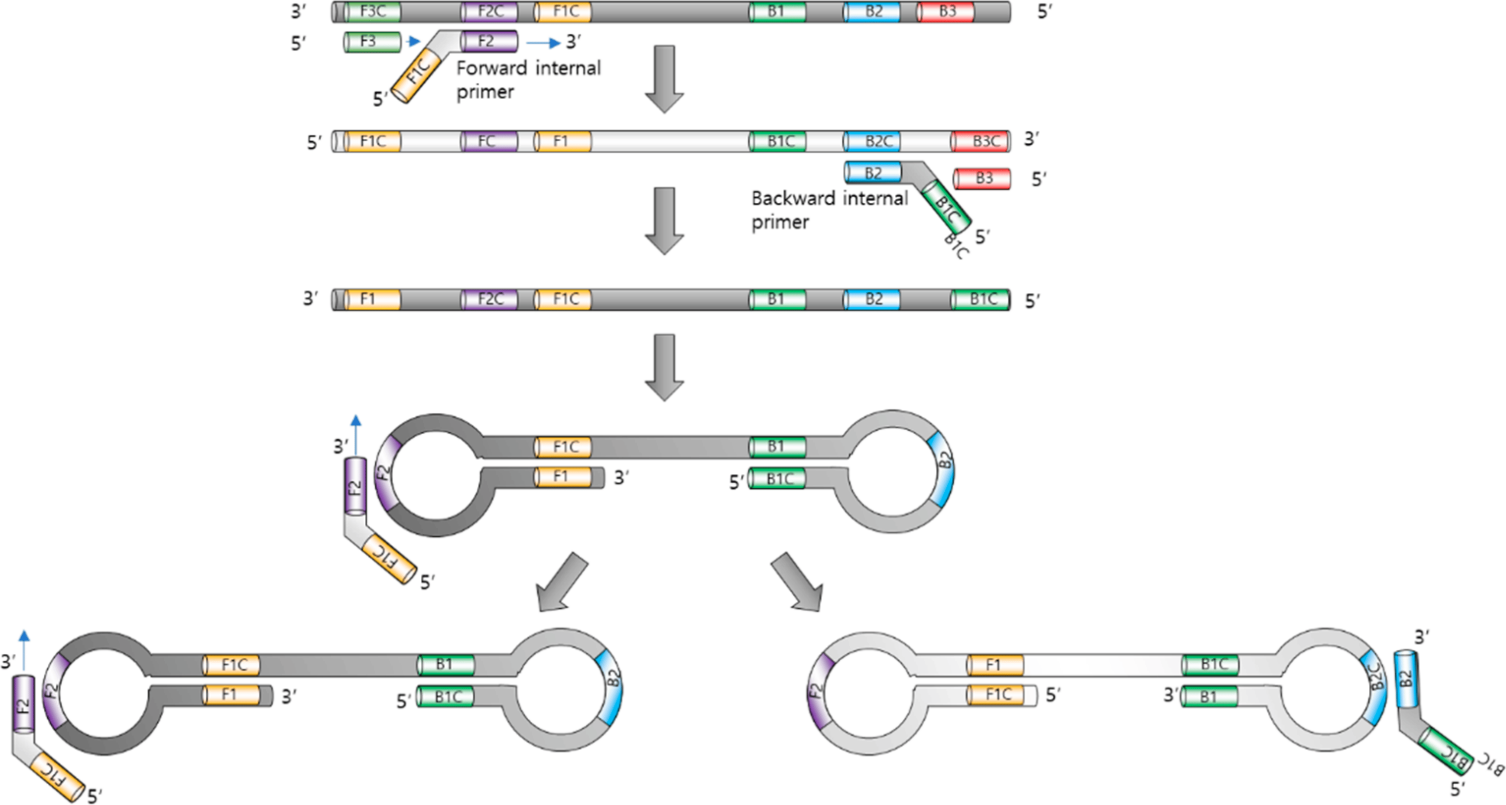
Loop-mediated isothermal amplification. Adopted with permission from Park (2022).

The LAMP is widely recognized for its simplicity, high specificity and sensitivity, as well as the ability to rapidly increase target sequences without the need for expensive equipment, such as thermocycles (Soroka et al., 2021). The LAMP process uses several primers that target different areas within the nucleic acid sequence. These primers include internal primers (FIP and BIP), external primers (F3 and B3) and loop primers (LF and LB), which initiate DNA synthesis with displacing activity (Notomi et al., 2015) (**Figure 2**).

The reaction proceeds at a constant temperature (typically between 60°C and 65°C), eliminating the need for thermal cycling. This allows the use of simple equipment that does not require complex settings and temperature control, which makes the LAMP simple and suitable for in-field use (Zanoli and Spoto, 2012). The operating principle is based on the cyclic amplification of target sequences of DNA or RNA using specially developed primers and unique enzymes. LAMP begins with the external primers binding to the target DNA or RNA sequence. The inner primers then form loops at complementary sites and initiate the synthesis of new strands. This produces characteristic D-loops and stem-loop structures (Park, 2022). As a result, the display of the synthesis of new strands continues cyclically, resulting in an exponential increase in the specific target sequence.

RT-LAMP assay was developed recently for detection of *Yam mosaic virus* using newly designed YMV1-OPT primers (Festus et al., 2023). The assay detected 0.1 fg/µL of purified RNA with a sensitivity equivalent to RT-PCR, developed in the same study. In contrast, other similar studies claimed that RT-LAMP had a higher sensitivity and specificity than the RT-PCR method (Hua et al., 2023; Kimura et al., 2023). In another study, Caruso et al. (2023) developed the design of six LAMP primers that showed high level of efficiency, sensitivity and selectivity to detect *Tomato leaf curl New Delhi virus*. Notably, this assay showed 1000 times more sensitivity than traditional PCR with comparable specificity. An important feature of LAMP is that it can be successfully carried out using a crude extract of an infected plant (Panno et al., 2020). This in turn would reduce the overall assay performance time as well as making it more efficient for development of a potential on-site detection tool due to its simplicity of sample preparation.

The RPA is an isothermal method of nucleic acid amplification, which is used for the rapid and specific diagnosis of various infectious diseases, including viral infections in plants. The system operates at a constant temperature (usually between 37-42°C) and does not require complex equipment, which makes this method especially useful for use in field conditions and/or in resource-limited environments. The RPA process is based on the use of recombinant proteins that recognize and bind to the target DNA. Recombinant proteins (recombinases) bind to primers, then recombinase-primer complexes are recognized and bind to complementary sequences on the target DNA. They are embedded in the target DNA, replacing the original chains. Stabilizing proteins protect the single-stranded sections of DNA that are formed. After that, the polymerase synthesizes a new DNA chain starting with primers, which leads to an exponential increase in the number of copies of the target sequence (Ivanov et al., 2021). The method is rapid operating at a constant temperature, which simplifies the technical equipment. It can detect low amounts of target DNA or RNA, and specificity is provided by recombinant proteins and specific primers. It also could be used for multiplex detection such as the PCR method. For instance, an RPA analysis was developed to detect DNA and RNA viruses of *Cucurbit leaf crumple virus*, *Cucurbit yellow stunting disorder virus* and *Cucurbit chlorotic yellows virus* (Jailani and Paret, 2023). The assay time was 80 minutes with the ability to process several mixed plant DNA and RNA viruses simultaneously. RPA was also used for simultaneous detection of *Maize chlorotic mottle virus* and *Sugarcane mosaic virus* in maize (Gao et al., 2021). The detection limit of the RPA method was 102 copies/μL, while conventional PCR showed sensitivity 10 times less. The analysis was carried out in only 30 minutes and was specific for the simultaneous detection of two viruses, since no cross-reaction was detected with three other viruses infecting maize. In another study, fast and sensitive RPA was created in combination with analysis using a lateral flow dipstick (LFD) to detect *Bean common mosaic virus* (Qin et al., 2021). The sensitivity of this RPA-LFD assay was 1000 times higher than conventional PCR assay and no cross-reaction was detected with other *Potyviruses*.

In comparison with other conventional diagnostic methods, LAMP can amplify DNA at detectable levels within 30-60 minutes, which is significantly faster than any PCR-based approaches. RPA is even faster, often achieving results in only 10-20 minutes (Bhat et al., 2022). In terms of sensitivity, isothermal methods can achieve detection limits that are comparable to, if not better than, those of PCR. For instance, LAMP can detect a few to several tens of copies of a target sequence, making it highly sensitive. However, isothermal amplification is limited by the species level, while primers have been developed for the PCR method can detect viruses at various taxonomic levels (Rubio et al., 2020). In contrast, their ease of use and minimal equipment requirements make them particularly attractive for field diagnostics and use in resource-limited settings. However, the choice of method should be guided by specific diagnostic needs, considering factors such as speed, simplicity, and sensitivity.

## 3 Biosensor technologies

Biosensors are innovative tools that have been developed with the purpose of determining the presence and quantification of target biological components within testing samples. The working principle of biosensor techniques is based on the ability of biological elements such as antibodies or nucleic acids to bind specifically to targeted analytes. This interaction changes any physical or chemical properties, which can then be measured by a sensor (Naresh and Lee, 2021). These devices provide multiple advantages including exceptional performance, possibility to integrate natural or synthetic antibodies, rapid response, high sensitivity and specificity, portability, miniaturization capability, and real-time analysis (Saylan et al., 2019; Dyussembayev et al., 2021). In addition, they offer an affordable and accessible means to swiftly detect plant pathogens in-field. In fact, most of the infectious agents detected by biosensors are human pathogens, including *Human immunodeficiency virus* (Babamiri et al., 2018), Hepatitis B (Tam et al., 2017), Ebola infections (Baca et al., 2015), *Norovirus* (Hwang et al., 2017). However, over the previous two decades, biosensors have been employed in a wide range of applications including detection of plant viruses such as *Cowpea mosaic virus*, *Tobacco mosaic virus*, *Salad mosaic virus* (Fang and Ramasamy, 2015).

**Figure 3:**
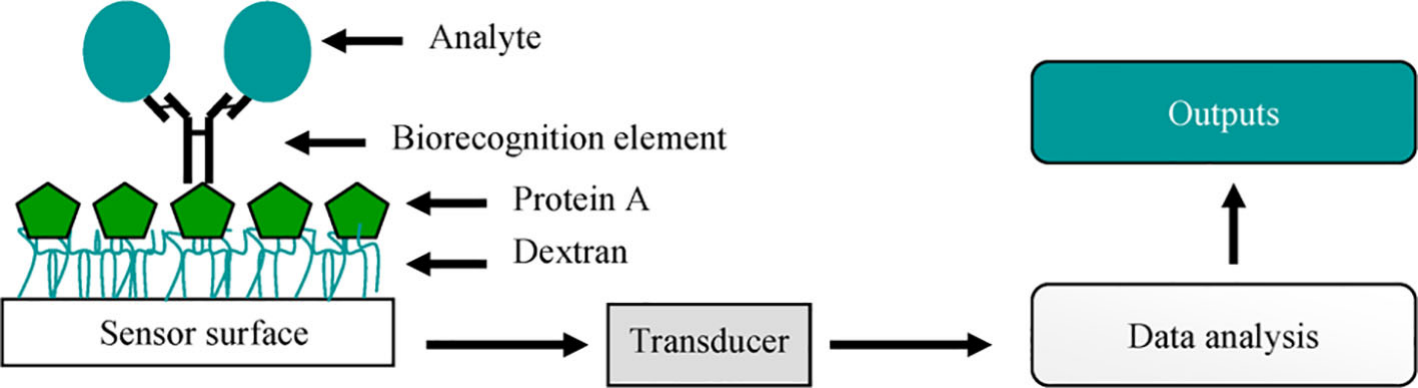
A simple representation of a biosensor. Adopted with permission from Byrne et al. (2009).

Biosensors are usually classified according to their signal transduction and biorecognition principles. The transduction system can include electrochemical, optical, piezoelectric, and thermal sensors (Martinelli et al., 2015). However, the biosensors are more often divided into two large groups according to their biorecognition elements: antibody- and nucleic acid-based biosensors. The antibody-based biosensors have distinctive signs of binding antibodies to target molecules, which ensures high detection accuracy (**Figure 3**). These antibodies can be directly attached to the surface of the sensor or to contacts with magnetic beads for immunomagnetic separation and subsequent detection (Byrne et al., 2009; Patel, 2021).

A method for detecting the *Maize chlorotic mottle virus* has been developed using a quartz crystal microwave (QCM) immunosensor with a special gold piece (Huang et al., 2014). The assay includes using quartz microweights, which can change their frequency depending on the mass adsorbed on their surface. This frequency change is used to quantify the presence of the virus. Gold surfaces of QCM crystals serve as the basis for subsequent binding of antibodies. As a result, the detection limit was approximately 250 ng/mL, which is comparable to the existing ELISA method. The QCM sensor has shown high specificity and sensitivity to both purified *Maize chlorotic mottle virus* and crude extracts from maize leaf samples. Another type of biosensor based on surface plasmon resonance system employing monoclonal antibodies was developed to detect *Potato virus Y*. The ability of the biosensor to detect and quantify *Potato virus Y* particles was compared with ELISA and RT-qPCR. The detection limit of the biosensor assay was 0.31 mg/mL, which is much less sensitive than the 0.019 mg/mL of ELISA (Gutiérrez-Aguirre et al., 2014). A biosensor utilizing surface plasmon resonance was used for the specific detection of *Maize chlorotic mottle virus* (Zeng et al., 2013). In lower concentrations assay enabled the detection in 30 minutes that faster than ELISA assay and with a detection limit of approximately 1 ppb (parts per billion). A similar surface plasmon resonance biosensor has been successfully developed for detection of the *Potato virus Y*, *Apple stem pitting virus*, *Maize chlorotic mottle virus*, *Barley stripe mosaic virus*, and *Cowpea mosaic virus* (Hong and Lee, 2018). The bioelectronic biosensor has been developed based on an electrolyte-gated organic field-effect transistor for the specific detection of *Plum pox virus* in *Nicotiana benthamiana* plant extracts (Berto et al., 2019). The working principle relies on specific binding of viral particles to anti-viral antibodies in plant extracts with a sub ng/mL detection limit. Notably, the authors claimed that the novel electronic sensor could potentially be manufactured using cost-effective methods and easily adapted into a portable device suitable for use in field conditions.

In contrast, nucleic acid-based biosensors represent another important class of analytical tools. Particularly, DNA biosensors are mainly used for diagnostic purposes and the principle is often based on a simple hybridization process where DNA probes bind specifically to target complementary sequences, allowing the detection and quantification of specific target sequences through a DNA (Carvajal Barbosa et al., 2021). Briefly, in the presence of targeted analyte, hybridization occurs between the DNA probe and the analyte, resulting in altered physical or chemical properties of the sensor. These DNA-based biosensors, also called genosensors, have been used for plant viral diagnostics. For example, Malecka et al. (2014) proposed a DNA biosensor for detection of specific oligonucleotides sequences of *Plum pox virus* in plant extracts. The sensor demonstrated a selectivity in discriminating between healthy and infected plants with a detection limit of 12.8 pg/mL.

Another example of DNA biosensor is QCM-based piezoelectric sensor based on DNA/RNA hybridization developed by Eun et al. (2002) for detection of two orchid viruses: *Cymbidium mosaic virus* and *Odontoglossum ring spot virus*. The specific nucleotide probe-coated DNA sensor was able to detect both viruses in quantities as low as approximately 1 ng in purified RNA preparations and 10 ng in the crude sap of infected orchids. In general, nucleic acids are often chosen as a tool to mediate various physico-chemical interactions in biosensors due to their specificity and sensitivity. This allows nucleic acids to interact more accurately and efficiently with target molecules than protein components such as antibodies. Thus, nucleic acid-based biosensors can detect significantly lower concentrations of target molecules, making them more sensitive and therefore potentially more useful in various applications where high precision and sensitivity are required (Hong and Lee, 2018).

DNA Microarray biosensors have been also developed for detection of several plant viral pathogens (Zherdev et al., 2018). This method is based on the use of virus-specific oligonucleotides that bind to a membrane or glass. After the total RNA is converted to cDNA and increased by PCR using pathogen-specific primers labeled with markers suitable for detecting molecules, the amplified and labeled products are applied to the array and DNA hybridization process is performed. After cleaning, the array will show the result depending on the marker used (Bhat et al., 2020).

A DNA microarray has been developed by Krawczyk et al. (2017) and it is designed for simultaneous identification of five pathogens of maize: *Pantoea ananatis*, *Pantoea agglomerans, Enterobacter cloacae* subsp., *Maize dwarf mosaic virus* and *Sugarcane mosaic virus*. Two more similar assays have been effectively developed for simultaneous detection of various potato viruses (Bystricka et al., 2003; Agindotan and Perry, 2008). Generally, comparing microarray-based and other biosensors in plant viral diagnostics, it can be noted that microаrrays can simultaneously analyze many molecules, which increases the effectiveness of pathogen screening (Govindarajan et al., 2012). While other biosensors are usually easier to use and require a smaller sample volume, which makes them more convenient for field research. However, the combination of their advantages would lead to even more effective and innovative diagnostic platforms.

## 4 Sequencing-based diagnostics

Several sequencing-based diagnostic techniques have been applied for detecting plant viruses, including next-generation sequencing. These approaches allow the detection and identification of viral pathogens in various plant samples (Pecman et al., 2017). By sequencing the entire nucleic acid content in the sample, researchers can simultaneously detect known and new viruses, identify genetic variants and characterize viral populations in the sample (Qin, 2019). One of the main advantages of sequential diagnostics is their ability to provide comprehensive information about the virus community present in the sample (Hadidi et al., 2016), unlike traditional diagnostic methods that target specific viruses or virus families (Avila-Quezada et al., 2022).

Sequencing-based technologies are widely used for detecting plant viruses due to high performance, sensitivity and scalability. In addition, high-throughput sequencing allows for a faster transition from virus discovery to the development of specific detection methods such as PCR or LAMP. It also contributes to the improvement of existing methods by identifying sequence variations within viral populations (Maree et al., 2018). These platforms allow researchers to quickly and cost-effectively generate massive sequences of action data, facilitating large-scale viral surveys, epidemiological research and outbreak investigations.

### 4.1 Next-generation sequencing

Next-Generation Sequencing (NGS) refers to advanced sequencing technologies that allow for rapid and high-throughput sequencing of DNA or RNA (Qin, 2019). With this new generation of sequencing methods, it is possible to obtain detailed information about the genetic composition of viruses in a timely and accurate manner, which makes them a valuable tool in the fight against infectious diseases (Nafea et al., 2024). By overcoming the limitations of traditional methods, NGS provides more accurate and complete analysis. In plant virology, NGS has been instrumental in discovering and isolating numerous plant viruses such as the *Pepino mosaic virus* (Prabha et al., 2013), *Grapevine leafroll-associated virus 1* (Morán et al., 2023), *Citrus leaf blotch virus isolate mulberry alba 2* (Chen et al., 2022), *Cherry mottle leaf virus*, *Cherry virus A* (Rott et al., 2017) etc.

Apart from the discovering of new viruses, NGS can also detect complete nucleotide sequences of viruses. For example, a complete sequence of the *Artichoke latent virus* was obtained, and it identified the virus to the genus *Macluravirus* (Minutillo et al., 2015). Consequently, this allowed refutation of the fact that *Artichoke latent virus* was originally proposed as a member of the genus *Potyvirus*. In fact, it is important to not only detect known viral isolates but also ensure that the diagnostic assay is reproducible across testing viral population. For example, NGS analysis was able to identify 14 distinct isolates of *Apple stem spot virus* and five variants of *Apple leaf chlorotype virus* (Singhal et al., 2021). Similarly, seven species of plant viruses have been detected using metagenomic analysis via NGS in symptomatic and asymptomatic tea plants collected from the field, along with two novel viral species that were latent pathogens: *Tea plant necrotic ring blotch virus* and *Tea plant line pattern virus* (Hao et al., 2018). Mwaipopo et al. (2021) used NGS technology to identify common bean viruses in wild plants, and as a result, a wide range of virus species has been identified due to high throughput of NGS. In addition, RT-PCR was used for validation of results, and the study identified viruses from at least 25 different genera, highlighting the complexity of virus interactions within the studied ecosystems.

Among the various sequencing technologies, there are three main approaches: Illumina, Ion Torrent and PacBio. Each of these technologies has advantages and operating principles, making them suitable for different research tasks. Illumina is ideal for research requiring high precision and performance, especially for short DNA fragments. Ion Torrent is a more accessible and faster method, which makes it attractive for routine sequencing where high accuracy is not critical, while PacBio offers the unique advantage of long reads, which is important for high-complexity genome research, including the detection of structural variations and the assembly of new genomes. All these techniques have been used for studying various plant viruses (**Table 3**).

**Table 3:** Next-Generation Sequencing technologies used for plant viral diagnostics.

Technique	Pathogen	Main advantages	Read length	References
Illumina	*Carrot yellow leaf virus*, *Grapevine redblotch virus* *Citrus leprosis viroid* *Tomato apical stunt viroid* *Barley yellow dwarf virus* *Sugarcane yellow leaf virus*	High throughput Low error rate Cost effectiveness	100-300 bp	Villamor et al. (2019) Pecman et al. (2022) Lee et al. (2023)
Ion Torrent sequencing	*Citrus tristeza virus* *Tomato mottle mosaic virus* *Little cherry virus 1*	Cost effectiveness	200-400 bp	Fillmer et al. (2015) Katsiani et al. (2018) Bester et al. (2021)
Pacific Biosciences (PacBio) sequencing	*Cryphonectria hypovirus 1* *Barley yellow dwarf virus-GAV*	Long reads High accuracy Reproducibility	10-20 kb	Shen et al. (2020) Leigh et al. (2021)

### 4.2 Portable nanopore sequencing

**Figure 4:**
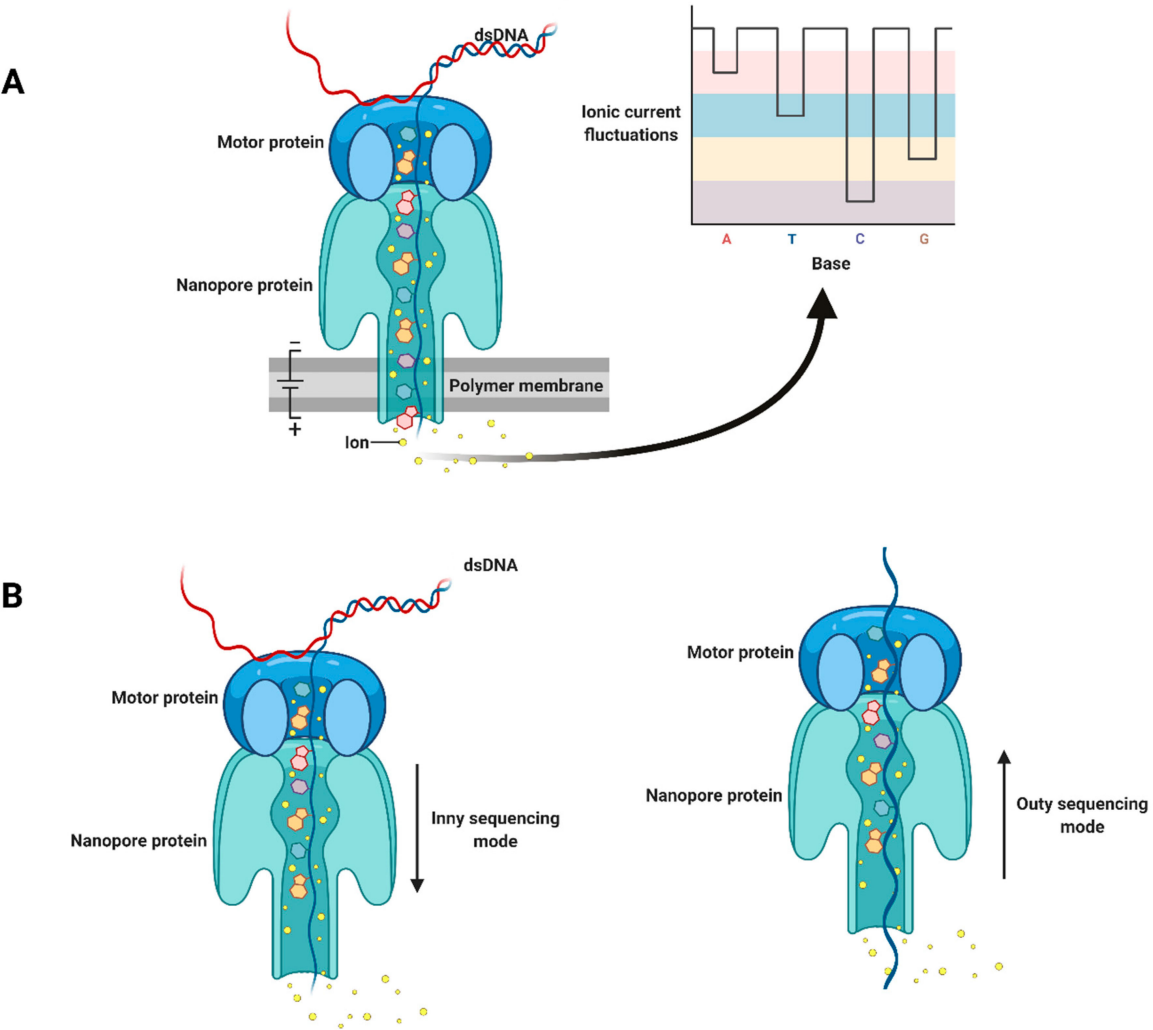
Schematic illustration of nanopore sequencing technology: **(A)** double-stranded DNA is unwound by a motor protein; **(B)** inny and outy sequencing modes. Adapted with permission from Javaran et al. (2021).

Portable nanopore sequencing technology has great potential to bring plant viral diagnostics to a whole new level by offering fast and accurate early diagnosis of viral pathogens directly in the field or at the point of need. It also provides rapid and efficient DNA/RNA sequencing, which makes it significantly advantageous over most conventional diagnostics (MacKenzie and Argyropoulos, 2023). The principle of sequencing technology is based on the transmission of single-stranded DNA or RNA through microscopic nanopores. During the process of passing through the nucleotides in the sample, the nucleotides act on the electrical field, resulting in changes in the current. These changes in the current can be fixed and analyzed by computer software to determine the sequence of nucleotides (Sun et al., 2022) (**Figure 4**).

MinION technology developed by Oxford Nanopore Technology (ONT) (Maliogka et al., 2018) is a portable single-molecule genome sequencing device (Bronzato Badial et al., 2018). The platform sequences both short and long reads as well as detect modified bases (i.e. methylation) in both DNA and RNA in real time (Garcia-Pedemonte et al., 2023). Javaran et al. (2023) recently used ONT based on direct-cDNA sequencing from dsRNA to detect multiple grapevine viruses. As a result, it was possible using this technology to detect as low concentrations of 20 common viruses as standard Illumina MiSeq sequencing was capable, which confirms its reliability and effectiveness. The ONT has also been used to detect a few yam viruses such as *Dioscorea bacilliform virus*, *Yam mild mosaic virus* and *Yam chlorotic necrosis virus* (Filloux et al., 2018). In another study, MinION was used for identification of *Cucumber Bulgarian latent virus*, which threatens cucumbers grown in greenhouses (Dong et al., 2022). Although MinION sequencing and real-time analysis using ONT EPI2ME WIMP reduced the assay performance time to 48 h, the read accuracy was low – around 83-98% identical to the reference genome. Also, too many gaps make the reads impossible to assemble. Consequently, this technology is still not as accurate as Sanger sequencing when it comes to analyzing viral whole genome sequences. Phannareth et al. (2020) performed detection of *Plum pox virus* in tobacco using MinION technology with two different kits: cDNA PCR Sequencing kit (SQK-PCS108) and Direct RNA Sequencing kit (SQK-RNA001). The results demonstrated the effectiveness of both kits in identifying plant viruses in tobacco samples. Moreover, for rapid identification of *Jasmine virus H* in *Ixora coccinea* plants, complete genome sequencing was performed using the ONT MinION platform only within 48 hours (Kim et al., 2023).

Compared with the other sequencing platforms, Nanopore sequencing can acquire entire viral genomes without assembly algorithms, thereby minimizing errors. Nanopore sequencing is widely available and allows long-read sequencing, and the simplicity of these long-lasting sequencing systems makes these devices attractive (MacKenzie and Argyropoulos, 2023). This technology allows direct sequencing of DNA or RNA samples and provides fast and real-time dynamic monitoring of sequencing data in the field (Chen et al., 2023).

### 4.3 CRISPR-Cas

The CRISPR-Cas system, discovered as part of the adaptive immune response of bacteria to viruses, has become an effective tool for genetic engineering as well as for plant pathogen diagnostics. Recently, it has also found application in the detection of plant viruses, offering accurate, sensitive and fast assays for diagnosing viral infections. Various approaches have been used to analyze nucleic acids, including Specific High-sensitivity Enzymatic Reporter Unlocking (SHERLOCK) and DNA Endonuclease-Targeted CRISPR Trans Reporter (DETECTR). SHERLOCK combines CRISPR-Cas 13a with isothermal amplification to detect specific RNA or DNA sequences of plant viruses, when DETECTR is based on CRISPR-Cas12a and targets DNA endonuclease (Fang et al., 2023). The basic principle is that the CRISPR-Cas system can be programmed to specifically detect by binding to specific sequences within viral genomes. When the CRISPR-Cas system detects a virus, a cascade of reactions is activated that effectively mark the viral sequence or even inactivate the virus (Cao et al., 2020; Lou et al., 2022).

Marqués et al. (2022) used CRISPR-Cas12a and CRISPR-Cas13a/d systems to detect three different viruses - *Tobacco mosaic virus*, *Tobacco etch virus*, and *Potato virus X* in tobacco plants. The results demonstrated that the use of these systems makes it possible to carry out simultaneous detection of multiple viruses accurately and quickly, which is important for on-site use. Another simple and fast diagnostic test was developed using CRISPR/Cas12a for the simultaneous detection of four RNA viruses and one viroid in apple (Jiao et al., 2021). Aman et al. (2020) also demonstrated RT-RPA single-step diagnostic assay employing CRISPR/Cas12a system for the detection of *Potato virus X*, *Potato virus Y* and *Tobacco mosaic virus*. The developed assay takes less than 30 minutes to perform and uses an inexpensive fluorescence visualizer, which makes it suitable for in-field diagnostics. Another CRISPR-Cas12a assay has been recently developed for detection of *Beet necrotic yellow vein virus* in sugar beet (Ramachandran et al., 2021). Notably, the assay involves RT-PA that allows the amplification of viral RNA at a constant temperature. This simplifies the process and eliminates the need for complex equipment. At the same time, the assay used a minimum number of primers as well as showing high level of sensitivity.

In general, CRISPR-Cas systems have not only been used in the diagnosis of plant viruses, but also been utilized for antiviral purposes in plants. CRISPR-Cas9 system has been shown promising results in conferring resistance to plant DNA and RNA viruses. The study by Ali et al. (2015) showed that the system introduced mutations into the target sequences of the *Tomato yellow leaf curl virus*. There was applied systemic delivery of sgRNAs aimed at coding and non-coding sequences of the virus. As a result, tobacco plants expressing CRISPR/Cas9 showed a decrease in the accumulation of viral DNA, which led to the elimination or significant reduction of infection symptoms. Recently, it has been developed CRISPR/Cas9-based banana genome editing, which can be used to create disease-resistant varieties (Tripathi et al., 2021). Similar strategy has also been used for inactivation of endogenous *Banana streak virus* by editing virus sequences (Tripathi et al., 2019). The results showed that multiplexing CRISPR/Cas9 technology is very effective for creating precise deletions in banana genome. Seventy-five percent of the edited events remained asymptomatic compared to the unedited control plants under water stress, which confirms the inactivation of the virus into infectious viral particles.

CRISPR-Cas-based pathogen detection systems have been attributed several advantages. Overall, they show great potential for on-site diagnostics due to their high sensitivity and specificity, and minimal requirement for advanced equipment (Wang et al., 2020; Venbrux et al., 2023). The system detects viral RNA without reverse transcription or amplification, which means the process becomes simpler, faster and more cost-effective (Devi et al., 2024). Traditional methods of virus diagnosis often require a long time to obtain results, while CRISPR-Cas analysis detection of the viral genome takes within a few hours. Moreover, it is possible to create tests based on paper strips using CRISPR-Cas that allow to quickly and conveniently determine the presence of viruses in plant tissues even without special equipment (Tsou et al., 2019).

## 5 Commercially available devices

Commercially available devices for the diagnosis of plant viruses are an invaluable tool for agronomists, gardeners and biologists. They provide the ability to quickly and accurately detect viruses in real time, which allows to quickly take measures to control the spread of infection. For the rapid detection of targeted analytes, tests are usually carried out using paper test strips, side-flow and vertical-flow immunoassay (Singh et al., 2018).

Paper sensors are considered as a new alternative technology that are being used as simple, inexpensive, portable and disposable analytical devices for many applications, including clinical diagnosis, food quality control and environmental monitoring. Furthermore, only a small number of samples and reagents are required for analysis on the paper substrate, making it ideal for application in disease diagnostics (Chailapakul et al., 2020). Typically, paper substrates are used that are pre-processed to apply bioreceptors or chemical reagents specifically related to the targeted analytes. The sample containing the analyte is applied to a paper strip, after which it migrates through the capillaries along the surface of the paper. During the migration of the sample, there is an interaction between the bioreceptors on the paper surface and the target analytes. This interaction leads to the formation of analyte-bioreceptor complexes that can be detected both visually and with special equipment (Yao et al., 2022).

Another example of commercially available diagnostic devices is a lateral flow immunoassay method (LFA), which is a rapid and simple test that can be carried out in field conditions. This method is based on the interaction of antigen and antibody, resulting in the formation of a visible signal, usually in the form of a color band, making it convenient to use (Byzova et al., 2018). LFA stripes have been developed that can distinguish *Tobacco mosaic virus*, *Tobacco vein banding mosaic virus* and *Potato virus Y* from mixed infection samples within minutes. The developed LFA strip can be widely used for the diagnosis of pathogens in the field as it already demonstrated the ability to accurately identify different strains of *Potato virus Y* (Guo et al., 2022). Other studies also pointed out the efficiency of this technique for accurate and rapid detection of *Grapevine leafroll-associated virus* (Byzova et al., 2018) and *Banana bract mosaic virus* (Selvarajan et al., 2020).

Agristrip technology is based on immunochromatographic assay principles, commonly known as lateral flow immunoassays utilizing monoclonal antibodies that are highly specific to the pathogen. Multiple detection methods such as Agristrip, microscopy, ELISA, PCR and PCR revealed infection of S. subterranea (Bouchek-Mechiche et al., 2011). The Agristrip developed for S. subterranea detection was as sensitive as DAS-ELISA, with a detection limit of 1-10 sponsors per mL of buffer. Also, Sss AgriStrip provides results within a very short timeframe, typically starting to show bands after 1 to 2 minutes, with maximum intensity reached after about 10 to 15 minutes. This test allows for quick and easy on-site detection, making it suitable for routine identification of powdery scab symptoms on tubers. This rapid response is essential in field settings, where timely identification of infected tubers can significantly impact disease management strategies and prevent the spread of the pathogen. Its design allows for on-site testing, which is invaluable during potato inspections at farms. By facilitating quick and accurate detection, the Sss AgriStrip plays a vital role in protecting potato crops and ensuring the sustainability of potato production (Bouchek-Mechiche et al., 2011).

## 6 Challenges and future directions

With the development of technologies and methods in molecular biology, the level of accuracy, sensitivity and availability of diagnostics for plant viral diseases has increased significantly. Consequently, recent advances in the diagnosis of plant viruses represent a significant breakthrough, opening new opportunities to control and prevent viral epidemics in agriculture. Despite these achievements, there are several challenges that may affect the obtainment of accurate and reliable results. One of the main problems is working with crude extracts from plant tissues, which often contain various contaminants and inhibitors that may affect subsequent molecular analysis. Crude extracts typically contain nucleic acids of both host and virus, which may result in false positives. Moreover, the presence of polysaccharides, polyphenols and other secondary metabolites in plant tissues may hinder DNA recovery procedures, resulting in low yields and poor DNA quality. In addition, the diversity of plant virus genomes makes the nucleic acid extraction difficult. Plant viruses may contain single-stranded RNA, double-stranded RNA, or single-stranded DNA genomes, each requiring special extraction techniques to effectively isolate viral nucleic acids. Standard DNA extraction protocols often include long steps such as shredding plant tissues, cell lysis and nucleic acid purification. However, these techniques may not be appropriate for all plant species or virus strains, resulting in inconsistencies in the efficiency and reliability of DNA extraction. Furthermore, the choice of extraction method may affect both the sensitivity and specificity of subsequent diagnostic tests. Some extraction methods can selectively lead to co-purification of inhibitors or destruction of viral nucleic acids, resulting in false negative results or reduced sensitivity of the analysis.

In contrast, recent advances in sequencing technologies have revolutionized the field of plant virology, offering powerful tools for comprehensive and high-performance virus detection, characterization and surveillance. In fact, some biosensors are compact devices that can provide rapid on-site detection of plant viruses, making them ideal for point-of-care diagnostics. NGS, on the other hand, offers a comprehensive and high-throughput approach to plant virus diagnostics. By sequencing the entire viral genome present in testing samples, NGS can identify multiple viruses simultaneously and even detect novel or emerging viral strains. This deep sequencing capability in turn allows a detailed characterization of the plant virome. Moreover, integrating CRISPR-Cas systems into plant virus diagnostics presents exciting opportunities for targeted and precise virus detection and manipulation. Additionally, CRISPR-Cas systems can be utilized for genome editing to confer resistance to specific viruses in plants, offering a sustainable solution to combat viral infections. Each of these approaches offers unique advantages and capabilities, making them valuable assets in the field of plant virology.

Nowadays, one of the most important concerns of growers is the availability of portable diagnostic devices when it comes to combating plant pathogens including viruses, which will allow quicker measures to control plant diseases. In fact, this could substantially improve informed disease management overall. As technology continues to evolve, drones could also be used to further enhance their ability to detect and monitor the condition of plants (Abbas et al., 2023).

## 7 Conclusion

Economic losses are estimated to exceed several billion dollars per year worldwide due to lack of timely conducted management strategies. Plant diseases caused by viruses can be effectively controlled if control agents are used at the initial stage of the development of viral diseases or by planting virus-free crops. Therefore, rapid and accurate disease diagnostics is needed. Symptomatic diagnosis is still useful, but often provides erroneous results due to confusion related to the high variability of symptoms caused by interactions between the host and the virus or abiotic stresses. On the other hand, conventional lab-based detection methods require trained personnel and substantial amount of time to provide results. Therefore, reliable and portable diagnostic platforms are required that could be useful for timely decision making.
